# Association between Pain in Adolescence and Low Back Pain in Adulthood: Studying a Cohort of Mine Workers

**DOI:** 10.1155/2017/3569231

**Published:** 2017-03-06

**Authors:** David Jonsson, Lage Burström, Tohr Nilsson, Jens Wahlström, Hans Pettersson

**Affiliations:** ^1^Clinic for Spinal Cord Injuries, Rigshospitalet, University of Copenhagen, 2100 Copenhagen, Denmark; ^2^Department of Public Health and Clinical Medicine, Occupational and Environmental Medicine, Umeå University, 901 87 Umeå, Sweden

## Abstract

*Purpose. *To study the association of self-reported pain in adolescence with low back pain (LBP) in adulthood among mine workers and, also, study associations between the presence of LBP over 12-month or one-month LBP intensity during a health examination and daily ratings of LBP three and nine months later.* Methods. *Mixed design with data collected retrospectively, cross-sectionally, and prospectively. Data was collected using a questionnaire during a health examination and by using self-reported daily ratings of LBP three and nine months after the examination.* Results. *Pain prevalence during teenage years was 55% and it was 59% at age 20. Pain during teenage years had a relative risk of 1.33 (95% confidence interval 1.03–1.73) of LBP 12 months prior to the health examination, but with no associations with LBP intensity or LBP assessed by text messaging. Pain at age 20 years was not associated with any measure of LBP in adulthood. Daily ratings of LBP were associated with LBP during the health examination three and nine months earlier.* Conclusions. *There were no clear associations between self-reported pain in adolescence and LBP in adulthood. Self-reported daily ratings of LBP were associated with LBP from the health examination. Possible limitations for this study were the retrospective design and few participants.

## 1. Introduction

Low back pain (LBP) is a common health problem that imposes a considerable personal, community, and financial burden worldwide [1, 2]. A systematic review of population prevalence studies of LBP between 1966 and 1998 showed a point prevalence ranging from 12 to 33%, 12-month prevalence from 22 to 65%, and a lifetime prevalence ranging from 11 to 84% [3]. A more recent review concluded that the number of people suffering from LBP is likely to increase substantially over the next decades due to an aging population [1]. Most people will probably suffer from LBP at least once in their lifetime and many possibly for more than one period [4].

LBP is a symptom with different stages of impairment, disability, and chronicity [5]. In general, the pathophysiology for LBP is unknown, but known causes are osteoarthritis, inflammation in joints or supporting tissue, and herniated disc or trauma [6, 7]. Usually, LBP is caused by a self-limiting musculoskeletal disorder, which typically resolves itself within 8–12 weeks [8]. Episodes of LBP lasting less than three months (approximately 90% of cases) are usually benign and do not need specific treatment [5]. There are also milder episodes of LBP that last for only a few days, with musculoskeletal stiffness or diffuse back discomfort [9]. LBP is considered to be a fluctuating condition as an episodic and cyclic disorder, recurrent in a large proportion of cases and persistent in some [10, 11]. A literature review by De Vet et al. [4] suggested defining an episode of LBP “as a period of pain in the lower back lasting for more than 24 hours, preceded and followed by a period of at least one month without low back pain.”

Physical and psychosocial factors at work and lifestyle and individual factors have been implicated in the onset of symptoms [12, 13], and a previous history of LBP has been shown to be a possible predictive cause of future back problems [13, 14]. Studies have shown an association between LBP in adolescence and LBP in adulthood [14–19]. Since LBP is a fluctuating condition, the type of data collection method is of great importance in trying to identify and predict presence of pain, on a daily basis or over time, in the data collection.

The aim of this study was to investigate the association of pain in adolescence with LBP in adulthood in a cohort of mine workers. In addition, the relation of LBP assessed with daily text messages and LBP over the previous 12-month period was studied. Furthermore, the aim was to study the relationship between LBP assessed with text messages and previous one-month LBP intensity.

## 2. Materials and Methods

Data with information on pain in adolescence and LBP in adulthood was first collected through a questionnaire and a medical examination as part of a health examination in 2012. Three and nine months after the health examination, self-reported daily ratings of LBP were collected by the use of text messages. The daily pain ratings were assessed for 35 days in each period. The study design was a mixed method with data collected retrospectively, cross-sectionally, and prospectively.

The study population were employees at a copper mine in the northern part of Sweden. 153 of the total 374 (41%) workers of various occupational groups within the mining industry agreed to take part in the study. The majority of the participants were employed as truck drivers (30%), followed by the second and third largest occupational groups, loaders (22%) and outdoor laborers (12%).

The participants were asked to answer a questionnaire for supplementary background data such as age, occupation, exposure to agents in their occupation, stress, and smoking habits. Data on pain in different body locations during the 12 months before the health examination was assessed with the following question: “Have you experienced any discomfort (pain, inconvenience) in any of the following body regions the last 12 months: neck, shoulder, elbow, wrist/hand, upper back, lower back, hip, knee and foot/ankle?” The possible responses were “yes” or “no.” The variable for the prevalence of participants who experienced discomfort in the lower back in the previous 12 months before the health examination was designated as a 12-month prevalence of LBP. Data on the pain intensity during the previous month before the health examination in 2012 was assessed with the following request: “Grade the intensity of any symptoms you have experienced in the past month in the following body regions.” The same anatomical sites were used as described above. The response scale was None, Mild, Moderate, Severe, and Extreme. The variable for the pain intensity in the lower back among the participants the previous month before the health examination was designated as one-month LBP intensity.

Two physicians, specialists in occupational medicine, examined all participants and also interviewed them about earlier periods of back pain with the guidance of a structured form. Data used in this study came from the request: “Grade the worst experience of pain from the following periods of time. Indicate where the pain was localized.” The participants were asked to respond on a scale of 0–10 where 0 indicated no pain and 10 indicated the worst pain imaginable. The scale used to retrospectively grade the worst pain included on a time axis divided into preschool (up to the age of 6 years), school (8–12 years of age), teenage years (14–18 years of age), and from 20 to 60 years of age on a two-year range axis. The variables chosen to define adolescence were the value of the worst experienced pain from 14 to 18 years of age, hereby designated as teenage years, and the worst experienced pain at the age of 20, hereby designated as 20 years.

**Figure 1:**
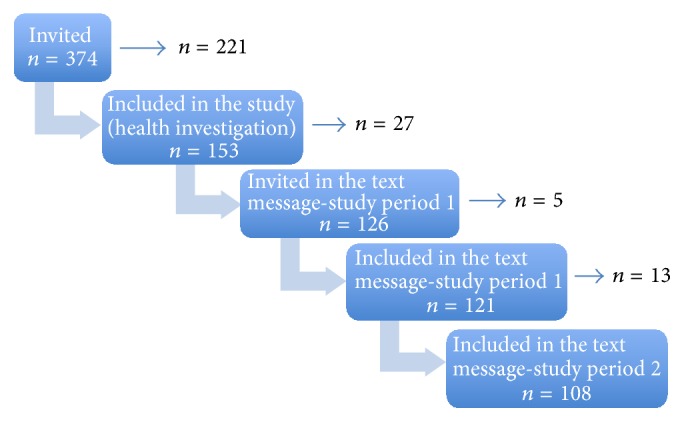
Flowchart of the invited subjects, inclusion, and those subjects who chose not to participate (arrow).

All the 153 employees that participated in the health examination were invited to participate in the text messaging study, which is described in more detail elsewhere [9]. No exclusion criteria were used. A flowchart of the invited subjects, inclusion, and the individuals who chose not to continue taking part in the measurements is shown in Figure 1. Eventually, 121 employees participated in the first period of data collection, which started on February 11, 2013, and continued until March 17, 2013. The second period started about six months later, September 9, 2013, and continued until October 13, 2013. Both data collection periods lasted for 35 days. During the data collection periods, the participants received a daily text message with the question “How much pain have you had in your lower back during the last 24 hours on a scale from 0 to 10? Reply with a number.” They reply with a numerical rating scale where 0 is “no pain” and 10 is “the worst pain imaginable.” The text messages were sent every day 15 minutes before the participant's daily shift ended. Answers received more than 24 hours after the question was sent were registered as missing [9]. Of the 121 participants, 108 (65 males and 43 females) fulfilled the second period of data collection. Reasons given for ending participation were vacation, not wanting to participate anymore, and end of employment. There were no significant differences between participants and dropouts in terms of gender, age, or pain rating (data not shown) [9]. The variable for pain in the lower back measured by text messaging was designated as text message (TM) first/second period.

Descriptive statistics were used to present characteristics of the participants at the health examination. For categorical data, relative risk with 95% confidence interval (95% CI) was used to study the associations of pain in adolescence and LBP in adulthood.

The participants were stratified in two groups according to their reported occurrence of pain in adolescence. The group categorized as “no pain” had indicated 0 on the pain scale, and those categorized as “suffering pain” had indicated 1–10 on the numerical pain rating scale. The association between pain in adolescence with one-month LBP intensity, 12-month prevalence of LBP, and the text message ratings of LBP was analyzed with variance analysis using ANOVA.

In the analyses of a possible association between one-month LBP intensity and pain in adolescence the one-month LBP pain intensity was dichotomized in a “low” (1 and 2) and a “high” (3–5) pain intensity group.

The text message ratings of LBP were calculated as the mean value of LBP for the 35-day measurement period, 3 and 6 months after the health examination. When studying the association of one-month LBP intensity with text message ratings of LBP, the one-month LBP pain intensity was categorized into four groups: “none” (1), “low” (2), “moderate” (3), and “high” (4 and 5). Pain groups 4 and 5 were merged due to the low number of subjects in the two highest categories.

Variance analysis using ANOVA was used to study associations between pain in adolescence with the text message ratings of LBP and also a possible interaction between gender and pain in adolescence. Variance analysis using ANOVA was also used to study the association between the 12-month prevalence of LBP and the one-month LBP intensity with the text message ratings of LBP.

Analyses were stratified by gender and by age. The participants' ages were dichotomized by the mean age of the participants, which was 40 years into a group consisting of participants 20–40 years of age and a group > 40 years of age. *p* value less than 0.05 was considered statistically significant. All analyses were performed using SPSS version 23 (IBM Corp., 2015).

The Regional Ethical Review Board for Medical Research in Umeå, Sweden, approved the study (2012-265-31M).

## 3. Results

Participant characteristics at baseline of the first text messaging period are shown in Table 1.

The prevalence of pain among all participants during the teenage years was 55% and at age 20 years it was 59%. It was more common among women and younger persons (20–40 years of age) to report pain in adolescence although this was not statistically significant (Table 2).

**Figure 2:**
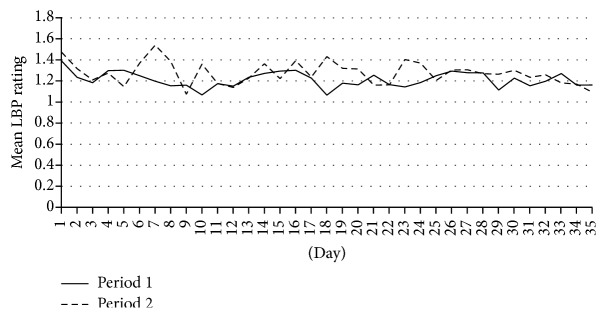
Mean daily rating during the first and second study period.

The mean LBP daily ratings by text messaging during study periods one and two are above one (Figure 2). Participants who experienced pain during their teenage years had relative risk of 1.33 (95% CI 1.03–1.73) of LBP during the 12 months' period preceding the health examination; however, there were no associations with one-month LBP intensity or LBP assessed with text messages in either of the two periods. Analyses stratified by gender showed an association with LBP assessed with text messages for the second period among the male participants (*p* = 0.034) (Table 3). ANOVA of a possible interaction between gender and pain in teenage years at the second period showed a significant interaction (*p* = 0.025) but not for the first study period (*p* = 0.076). Analyses defined by age divided into two age groups showed no statistically significant associations between back pain during the teenage years and LBP in the last 12 months, LBP intensity, or LBP as assessed by text messages (data not shown).

When analyzing all participants as one group, irrespective of gender, no statistically significant associations were observed between pain at the age of 20 years and the 12-month prevalence of LBP, LBP intensity, or LBP as assessed by text messages. Male workers who experienced pain at age 20 had a relative risk of 0.43 (95% CI 0.20–0.93) for LBP intensity (Table 4). ANOVA of a possible interaction between gender and pain in teenage years on LBP assessed with text messages at the first and second study period showed no significant interaction (*p* = 0.384 and *p* = 0.113, resp.).

The 12-month prevalence of LBP, as well as the one-month LBP intensity, was associated with mean LBP assessed with the text message-responses in both periods. With increasing one-month LBP intensity, the mean LBP rating increased (Table 5). ANOVA of a possible interaction between gender and LBP intensity on LBP assessed with text messages at the second study period showed a significant interaction (*p* = 0.017) and no interaction for the first study period (*p* = 0.332). The same interaction analysis of possible interaction between gender and the 12-month prevalence of LBP on LBP assessed by text messages showed no statistical significant interaction in the first or second study period (*p* = 0.312 and *p* = 0.353, resp.).

## 4. Discussion

The aim of this study was to investigate the association of pain in adolescence with LBP in adulthood in a cohort of mine workers. Only the 12-month prevalence of LBP among all participants and the intensity of LBP preceding the examination among males were associated with pain in adolescence. In our data, pain in adolescence was not associated with the presence or intensity of present LBP.

A possible limitation in this study might be the study design and the method used to collect information on back pain from adolescence. In previous studies that have shown associations, a longitudinal/prospective design has been used [15–19]. Harreby et al. [15] found that 90% of their study population, with history of LBP, had an increased 12-month prevalence of LBP when answering the questionnaire at the follow-up after 25 years. The study concluded that LBP might be influenced in many ways and that multiple risk factors seemed to be of importance. Hellsing and Bryngelsson [18] found that LBP at age 18 significantly increased the risk of LBP at age 40. In our study, 74% of the study population with a history of pain in adolescence and 50% of the study population with no history of pain in adolescence had a 12-month prevalence of LBP. The 12-month prevalence of LBP among those with pain in adolescence in our study was lower compared to Harreby et al. [15], but we had a retrospective design that may limit the details of earlier recollection of pain. The lack of significant associations in this study could also depend on low number of participants and therefore low statistical power. Earlier studies had many more participants ranging from 481 up to 6540 [14–19].

This study found a prevalence of pain during teenage years (14–18 years) of 55% and at age 20 years a corresponding prevalence of 59%. Hestbaek et al. [19] had a prevalence of LBP age 16–19 of 40% and at age 20–22 of 50%. LBP was defined as LBP for more than zero days of the past year. The prevalence of pain in our study is higher than that in Hestbaek et al. [19], but we asked for the worst pain in general and not specifically for back pain.

In the present study, the participants were asked to retrospectively report the occurrence of pain and rate its intensity. The relation between pain and time used a time axis divided into preschool, school, teenage years, and from 20 to 60 years of age in a two-year range. The mean age of the 121 participants was about 40 years, and almost half of the population studied were in the age category of 41–65 years (Table 1). A retrospective method, such as the one used, may result in imprecise data caused by the participant's inability to remember early periods of pain (i.e., recall bias), for some up to decades before data collection. As described by Hestbaek et al. [19], a total of 35% of their study population reporting LBP at baseline claimed never to have experienced LBP eight years later at follow-up. This recall bias might partly explain the results from the present study. During the analyses we divided the participants into two groups: 20–40 years of age and >40 years, to study whether the participants 20–40 years of age or those over 40 years of age ability to remember early periods of pain would be shown in the statistical analysis, but this did not significantly affect the results. The lack of connection between pain in adolescence and LBP in adulthood might depend on several factors occurring from younger age to older age which affect the LBP, such as other back traumas later in life. In this study the age distribution between males and females was different since the males were older than the females. This has not been taken into account during the analysis.

This study used two different data variables to define adolescence, one representing 14–18 years of age (teenage) and the other the age of 20 years. No mutual definition of adolescence/youth or pain prevalence has been observed in other studies investigating a possible association between back pain in adolescence and LBP in adulthood [15, 18–20]. The lack of mutual definition of adolescence/youth and pain prevalence between studies may explain difference in results. If only using the results from the 12-month prevalence of LBP and defining adolescence by using the category teenage years, that is, reducing the statistical variables in the analysis, the present study presented a result of a statistically significant association between pain in adolescence and LBP in adulthood (Table 3).

When handling prospective data such as observations of pain, these are often collected and analyzed at a few points of time, such as at baseline and at follow-up. If the measured outcome such as LBP has a tendency to fluctuate, details of relapses and remissions might be hard to detect only by two occasions of measurement [21]. It is then preferable to collect data on a daily basis. Collecting information on daily pain has often been conducted by diaries. Diaries make it possible to gather long-term information on the way individuals feel or spend their time on certain activities of relevance to a research project, for example, a change of symptoms over time or compliance with treatment [8].

Since the use of mobile phones is standard among people, an alternative method of data collection has become available in the form of text messages. By using this method, it has become possible to collect data on a monthly, weekly, daily, or even hourly basis [22]. In this study, the response rates from the data collection were very high, which indicates that the participants found the method easy to reply to, although there were 13 participants who did not participate in the second measurement period. End of employment and no interest in participating further were specified as reasons not to continue the measurements, which could indicate that the method was a burden to them [9]. The present study shows high response rates which are consistent with similar studies that used text messaging for participants with LBP [10, 21].

The second aim of this study was to study the association between the 12-month prevalence of LBP and a one-month LBP intensity rating before the health examination with self-reported daily ratings of LBP collected by text messages. The 12-month prevalence of LBP and one-month LBP intensity assessed at present were, as shown in Table 5, statistically significant associated with LBP assessed with the two measuring periods, for a total of 70 days, three months, and nine months subsequently. For one-month LBP intensity, the mean LBP rated by text messaging increased with the LBP intensity in both the first and the second period of measurement. There was also an interaction of gender and LBP intensity on LBP assessed by text messaging during the second study period. The self-reported LBP by text messaging provided detailed information in a fluctuating condition among the study population, and, as the participants only had a predetermined and limited time to answer, the method might be considered less vulnerable to recall biases compared to other methods. Questionnaire surveys are not considered ideal for collecting frequent data, due to often poor response rates. To improve response rates, several mailings are often needed, which will increase costs [21]. The cost of using text messages as data collection method is very low compared with postal questionnaires and also promising in terms of minimal time consumption and minimal data handling, for both researchers and participants [22]. This method of collecting data may be useful when measuring fluctuating conditions with considerable individual variation [10, 21, 22]. In our study we recorded fluctuating pain over time but we could not find a suitable quantification other than the mean value to account for this fluctuation.

To our knowledge no cohort of workers has yet been followed in order to study a possible association between pain in adolescence and LBP in adulthood. An earlier study has shown that using daily text messages to investigate the presence of LBP on a long-term basis is possible [9]. Previous published studies using text messages to study the prevalence of back pain have included individuals with known low back pain [10, 21–23]. Thus, if studying the risk factors for LBP in a working population, it is important to ask about back pain episodes in the immediate past, that is, maybe last year, since this is a strong predictor of subsequent LBP. However, it seems unnecessary to ask about pain in adolescence, since that is a measure most probably strongly influenced by recall bias.

## 5. Conclusions

No clear associations between retrospective information on self-reported pain in adolescence and LBP in adulthood were observed. Possible limitations for this study were its retrospective design and few participants. The result of this study indicates that a prospective longitudinal method is the preferred method when investigating associations between pain in adolescence and LBP in adulthood, when a retrospective method may give imprecise data caused by recall bias.

The occurrence of LBP in the 12-month period preceding the health examination and a high LBP intensity one month before the health examination was strongly associated with the mean LBP over the 35 days assessed by daily text messages three and nine months later.

In order to investigate a possible relation between pain in adolescence and LBP in adulthood, a prospective study should be used to ensure detailed information.

## Figures and Tables

**Table 1:** Description of the participants included in the first text message-study period, at baseline, in relation to gender, age, height, weight, BMI, and smoking status.

Characteristics	Participants (*n* = 121)		All
	Male	Female	
Gender *n* (%)	73 (60.3)	48 (39.7)	121 (100)
Age (years), mean (SD)	42.8 (10.3)	36.2 (10.6)	40.2 (10.9)
Category 20–40 years *n* (%)	28 (38.4)	34 (70.8)	62 (51.2)
Category 41–65 years *n* (%)	45 (61.6)	14 (29.2)	59 (48.8)
Height (cm), mean (SD)	178.3 (7.3)	165.7 (5.5)	173.3 (9.0)
Weight (kg), mean (SD)	85.5 (13.8)	68.2 (13.8)	78.6 (16.1)
BMI mean (SD)	26.8 (3.5)	24.8 (4.6)	26.0 (4.1)
Smoking (*n* = 120) *n* (%)			
Nonsmoker	51 (70.8)	28 (58.3)	79 (65.8)
Smoker	11 (15.3)	6 (12.5)	17 (14.2)
Former smoker	10 (13.9)	14 (29.2)	24 (20.0)

*n*: number of participants.

**Table 2:** Pain at teenage years and at age 20 years, by gender, age (categorized), and the relative risk (RR) with 95% confidence interval (95% CI) for gender and age groups.

	Pain teenage years^†^ *n* (%)		RR (95% CI)	Pain 20 years^†^ *n* (%)		RR (95% CI)
	Yes	No		Yes	No	
Gender						
Female	27 (57)	20 (43)	1.09 (0.78–1.51)	30 (68)	14 (32)	1.27 (0.94–1.71)
Male	38 (53)	34 (47)		36 (54)	31 (46)	
Age (categorized)						
20–40	37 (61)	24 (39)	0.80 (0.57–1.11)	37 (66)	19 (34)	0.80 (0.58–1.09)
>40	28 (48)	30 (52)		29 (53)	26 (47)	

*n*: number of participants.  ^†^Missing values.

**Table 3:** Association between pain in teenage years and mean values of one-month low back pain (LBP) intensity and 12-month prevalence of LBP, presented by gender. Relative risk (RR) with 95% confidence interval (95% CI) of pain in teenage years for one-month LBP intensity and 12-month prevalence of LBP categories. Variance analysis of pain in teenage years and the two periods of daily self-reported LBP by text message (TM) (mean value), presented by gender. *p* < 0.05 was considered statistically significant.

		Presence of pain in teenage years		RR (95% CI)
		Pain *n* (%)	No pain *n* (%)	
LBP intensity, female (*n* = 47)	High *n* (%)	12 (25.5)	9 (19.1)	0.99 (0.52–1.88)
	Low *n* (%)	15 (31.9)	11 (23.4)	
LBP intensity, male (*n* = 72)	High *n* (%)	13 (18.1)	11 (15.3)	1.06 (0.55–2.04)
	Low *n* (%)	25 (34.7)	23 (31.9)	
LBP intensity, all (*n* = 119)	High *n* (%)	25 (21.0)	20 (16.8)	1.04 (0.65–1.65)
	Low *n* (%)	40 (33.6)	34 (28.6)	
LBP 12-month prevalence, female (*n* = 46)	Yes *n* (%)	24 (52.2)	15 (32.6)	1.13 (0.86–1.47)
	No *n* (%)	3 (6.5)	4 (8.7)	
LBP 12-month prevalence, male (*n* = 71)	Yes *n* (%)	26 (36.6)	16 (22.5)	1.49 (0.99–2.26)
	No *n* (%)	11 (15.5)	18 (25.4)	
LBP 12-month prevalence, all (*n* = 117)	Yes *n* (%)	50 (42.7)	31 (26.5)	1.33 (1.03–1.73)
	No *n* (%)	14 (12.0)	22 (18.8)	

		Pain	No pain	*p* value

TM first period, female (*n* = 47)	Mean (SD)	1.68 (1.49)	1.24 (1.34)	0.304
		*n* = 27	*n* = 20	
TM first period, male (*n* = 72)	Mean (SD)	.78 (1.10)	1.24 (1.43)	0.126
		*n* = 38	*n* = 34	
TM first period, all (*n* = 119)	Mean (SD)	1.15 (1.34)	1.24 (1.39)	0.724
		*n* = 65	*n* = 54	
TM second period, female (*n* = 42)	Mean (SD)	1.68 (1.82)	1.10 (1.22)	0.246
		*n* = 23	*n* = 19	
TM second period, male (*n* = 64)	Mean (SD)	.83 (1.23)	1.58 (1.53)	0.034
		*n* = 34	*n* = 30	
TM second period, all (*n* = 106)	Mean (SD)	1.17 (1.54)	1.39 (1.43)	0.449
		*n* = 57		

*n*: number of participants.

**Table 4:** Association between pain at an age of 20 years and mean values of one-month low back pain (LBP) intensity and 12-month prevalence of LBP, presented by gender. Chi-square and Fisher's exact test of pain in teenage years and one-month LBP intensity and 12-month prevalence of LBP. Variance analysis of pain at an age of 20 years and the two periods of daily self-reported LBP by text message (TM) (mean value), presented by gender. *p* < 0.05 was considered statistically significant.

		Presence of pain at age 20 years		RR (95% CI)
		Pain *n* (%)	No pain *n* (%)	
LBP intensity, female (*n* = 44)	High *n* (%)	13 (29.5)	6 (13.6)	1.01 (0.49–2.10)
	Low *n* (%)	17 (38.6)	8 (18.2)	
LBP intensity, male (*n* = 67)	High *n* (%)	7 (10.4)	14 (20.9)	0.43 (0.20–0.93)
	Low *n* (%)	29 (43.3)	17 (25.4)	
LBP intensity, all (*n* = 111)	High *n* (%)	20 (18.0)	20 (18.0)	0.68 (0.42–1.11)
	Low *n* (%)	46 (41.4)	25 (22.5)	
LBP 12-month prevalence, female (*n* = 43)	Yes *n* (%)	27 (62.8)	9 (20.9)	1.30 (0.89–1.90)
	No *n* (%)	3 (7.0)	4 (9.3)	
LBP 12-month prevalence, male (*n* = 66)	Yes *n* (%)	19 (28.8)	19 (28.8)	0.83 (0.55–1.26)
	No *n* (%)	17 (25.8)	11 (16.7)	
LBP 12-month prevalence, all (*n* = 109)	Yes *n* (%)	46 (42.2)	28 (25.7)	1.07 (0.82–1.40)
	No *n* (%)	20 (18.3)	15 (13.8)	

		Pain	No pain	*p* value

TM first period, female (*n* = 44)	Mean (SD)	1.48 (1.25)	1.14 (1.26)	0.406
		*n* = 30	*n* = 14	
TM first period, male (*n* = 67)	Mean (SD)	.90 (1.39)	1.01 (1.18)	0.724
		*n* = 36	*n* = 31	
TM first period, all (*n* = 111)	Mean (SD)	1.16 (1.35)	1.05 (1.19)	0.655
		*n* = 66	*n* = 45	
TM second period, female (*n* = 39)	Mean (SD)	1.55 (1.74)	1.05 (1.07)	0.353
		*n* = 26	*n* = 13	
TM second period, male (*n* = 59)	Mean (SD)	.89 (1.27)	1.42 (1.64)	0.169
		*n* = 31	*n* = 28	
TM second period, all (*n* = 98)	Mean (SD)	1.19 (1.53)	1.30 (1.48)	0.719
		*n* = 57	*n* = 41	

*n*: number of participants.

**Table 5:** Variance analysis using mean values of one-month low back pain (LBP) intensity and 12-month prevalence of LBP, compared to the mean value of the two periods of daily self-reported LBP by text message- (TM-), presented by gender. *p* < 0.05 was considered statistically significant.

	Text message period 1				Text message period 2		
		Mean (SD)	*p* value			Mean (SD)	*p*-value
LBP intensity, female (*n* = 48)	None *n* = 10	1.05 (1.41)	0.001	LBP intensity, female (*n* = 43)	None *n* = 8	0.96 (1.68)	0.415
	Low *n* = 16	.72 (.63)			Low *n* = 14	1.16 (1.55)	
	Moderate *n* = 18	2.20 (1.61)			Moderate *n* = 17	1.87 (1.75)	
	High/very high *n* = 4	3.45 (1.30)			High/very high *n* = 4	2.17 (1.51)	

LBP intensity, male (*n* = 73)	None *n* = 32	.62 (.88)	<0.001	LBP intensity, male (*n* = 65)	None *n* = 27	.63 (.77)	<0.001
	Low *n* = 16	.75 (.68)			Low *n* = 13	.71 (.82)	
	Moderate *n* = 21	1.31 (1.29)			Moderate *n* = 21	1.82 (1.72)	
	High/very high *n* = 4	3.72 (2.31)			High/very high *n* = 4	3.15 (1.70)	

LBP intensity, all (*n* = 121)	None *n* = 42	.72 (1.03)	<0.001	LBP intensity, all (*n* = 108)	None *n* = 35	.70 (1.03)	<0.001
	Low *n* = 32	.74 (.65)			Low *n* = 27	.95 (1.25)	
	Moderate *n* = 39	1.72 (1.49)			Moderate *n* = 38	1.84 (1.71)	
	High/very high *n* = 8	3.58 (1.74)			High/very high *n* = 8	2.66 (1.58)	

LBP 12-month prev., female (*n* = 47)	No *n* = 7	.60 (.69)	0.058	LBP 12-month prev., female (*n* = 42)	No *n* = 5	.17 (.22)	0.049
	Yes *n* = 40	1.78 (1.56)			Yes *n* = 37	1.72 (1.69)	

LBP 12-month prev., male (*n* = 72)	No *n* = 29	.71 (.91)	0.089	LBP 12-month prev., male (*n* = 64)	No *n* = 24	.70 (.79)	0.026
	Yes *n* = 43	1.24 (1.45)			Yes *n* = 40	1.51 (1.61)	

LBP 12-month prev., all (*n* = 119)	No *n* = 36	.69 (.86)	0.004	LBP 12-month prev., all (*n* = 106)	No *n* = 29	.61 (.75)	0.002
	Yes *n* = 83	1.50 (1.52)			Yes *n* = 77	1.61 (1.64)	
